# Serological diagnosis of cysticercosis in humans and pigs: status, limitations, and prospects

**DOI:** 10.3389/fvets.2025.1558555

**Published:** 2025-07-15

**Authors:** Md. Shahadat Hossain, Shafqat Shabir, Nicholas Ngwili, Lian F. Thomas, Franco H. Falcone

**Affiliations:** ^1^Department of Parasitology, Bangladesh Agricultural University, Mymensingh, Bangladesh; ^2^Institute of Parasitology, Justus Liebig University Giessen, Giessen, Germany; ^3^International Livestock Research Institute, Nairobi, Kenya; ^4^Royal (Dick) School of Veterinary Sciences, University of Edinburgh, Edinburgh, United Kingdom

**Keywords:** cysticercosis, human, pig, *Taenia solium*, serological tests, IgE

## Abstract

Cysticercosis is a neglected zoonosis caused by *Taenia solium*, which involves pigs as intermediate hosts, leading to pig cysticercosis (PCC). Humans are the only definitive hosts, harbouring the mature tapeworm in the small intestines, but they can also act as intermediate hosts upon accidental ingestion of eggs, resulting in human cysticercosis (HCC), called neurocysticercosis (NCC) when the cysts lodge in the central nervous system. Diagnosis of HCC/NCC in humans is based on imaging technologies and serology. The gold standard method for PCC diagnosis is the full carcass dissection and recovery of cysts. However, tongue palpation and meat inspection are the most widely used methods in endemic countries. These methods are specific at the genus level but cannot distinguish mixed infection from other taeniids and are not sufficiently sensitive in pigs with low infection. Available serological tests for human and pig infection are based on parasite-specific immunoglobulin G (IgG). Still, most tests are either cross-reactive with other taeniids or not sensitive enough for single or inactive cysts, particularly for NCC patients. Here, we compare various serological techniques for PCC and NCC published since 2000 and discuss the benefit of IgE-based serodiagnosis as a potential alternative to traditional serology. Considering the diagnostic limitations described above and the need to identify endemic areas to prevent transmission between humans and pigs and monitor control efforts, the development of more sensitive and specific serological tests, followed by a field-applicable point-of-care (POC) test for cysticercosis, is of the utmost importance.

## 1 Introduction

Cysticercosis is a food-borne parasitosis caused by *Taenia solium* (*T. solium*) in humans and pigs. The World Health Organization (WHO) has identified cysticercosis as one of the twenty-one neglected tropical diseases (NTDs) worldwide (1), while the U. S. Centers for Disease Control and Prevention (CDC) has designated cysticercosis as one of the five neglected parasitic infections in the United States (2). This zoonotic parasite has been estimated to cause the highest number of foodborne Disability Adjusted Life Years (DALY) by the WHO Foodborne Disease Burden Epidemiology Reference Group (3), excluding viral and bacterial diseases (but including protozoan parasites). Furthermore, it is considered a significant public health problem in most endemic countries in Latin America, Africa, and Asia (4).

Human cysticercosis (HCC) is caused by the larvae of *T. solium*, which predominantly affect the central nervous system (CNS), accounting for 86% of cases (5). However, cysts may also develop in extra-CNS sites, including the eyes, muscles, skin, subcutaneous tissues, heart, lungs, and peritoneum (4, 6). These non-neural manifestations diaphragm are collectively referred to as extraneural HCC and can be further categorized into intramuscular, subcutaneous, cardiac, pulmonary, and ophthalmic forms (5, 7–11). The neural form of cysticercosis, known as neurocysticercosis (NCC), is a major neurological disorder in humans, estimated to account for up to 30% of avoidable epilepsy (12, 13). It evolves in endemic countries with low sanitation coverage conditions and a free-ranging pig production system. There are two types of NCC: Parenchymal NCC (occurring in the brain tissue) and extraparenchymal NCC (occurring in the intraventricular and subarachnoid spaces of the brain and spinal cord). Extraparenchymal NCC is the most severe form (14) and most resistant to drug treatment (15). Around 2.56–8.30 million people are affected by NCC, including symptomatic and asymptomatic cases (16). The larval/cysticercus stage of *T. solium* also causes pig cysticercosis (PCC). In pigs, *T. solium* larvae mostly remain in the host muscles but can also occur in other organs, including the eyes and tongue (17). This results in carcass condemnation and decreased value of pigs, with an estimated 20–60% production losses in pig-raising countries (18).

**Figure 1:**
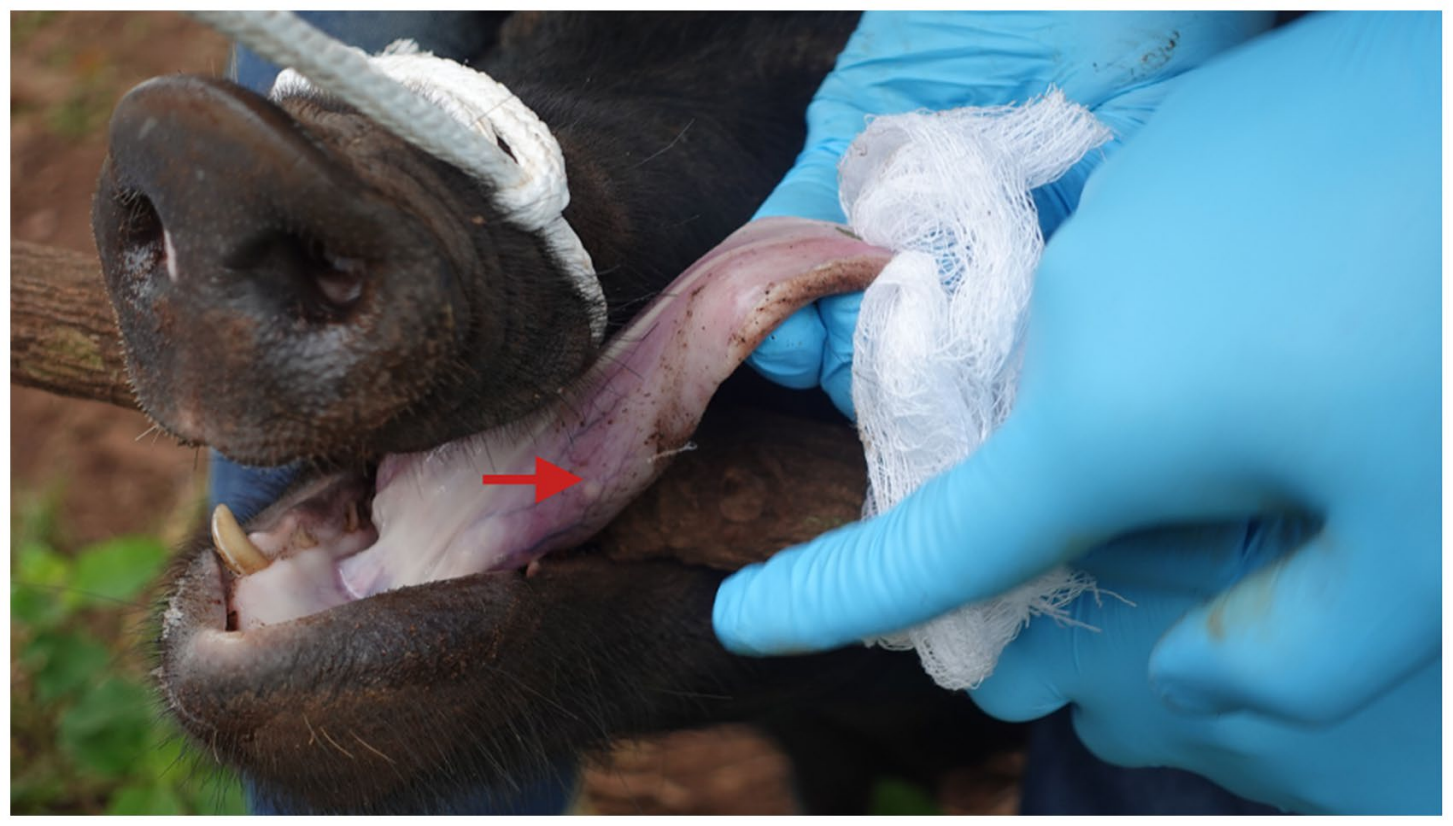
Tongue palpation of pig for testing cysticercosis in Uganda. The red arrow indicates a visible, palpable cyst. This method only works in heavily infected animals (photo: Md. Shahadat Hossain).

Diagnosing *T. solium* cysticercosis in humans and pigs depends on the organ affected and the severity of the infection. Additionally, the occurrence of other *Taenia* species (e.g., *T. saginata, T. hydatigena, T. asiatica*) in the same environment and cross-immunity among these species (19) could have moderated or modified the prevalence of *T. solium* in pigs and humans, as found in South East Asia and some African countries (20–22). This interspecific competition and cross-reactivity hinder the differential diagnosis of cysticercosis at the species level, leading to inadequate diagnostic capability and poor infection investigation. Therefore, in endemic countries where different *Taenia* species of pig (*T. solium*, *T. hydatigena, T. asiatica*) are prevalent, species-specific diagnosis for *T. solium* is difficult (22, 23). For NCC patients, a definitive diagnosis requires advanced neuro-imaging (magnetic resonance imaging [MRI] and a non-contrast computed tomography [CT] scan of the brain), brain or spinal cord biopsy, or in rare cases, visualization of subretinal cysts (24). HCC involving the intramuscular, cardiac, and pulmonary systems also requires imaging techniques as in NCC (25–27), whereas ophthalmic cysticercosis is mostly diagnosed using orbital ultrasonography (28). However, neuro-imaging techniques are expensive and often unavailable in endemic areas where tapeworm infection is frequent (29). For the diagnosis of PCC, the most widely used and cheapest methods are tongue palpation (Figure 1) and routine meat inspection of pigs (30, 31). Tongue palpation specificity is close to 100%, but its sensitivity is as low as 16.1% (95%CI: 5–34) (32), thus detecting only heavily infected pigs. Routine meat inspection showed high specificity (100%) with slightly higher sensitivity - 38.7% (95%CI: 22–58) than tongue palpation, but it is also insensitive in light infections (32). The gold standard method for diagnosing PCC is complete carcass muscle/brain dissection, as it has shown greater sensitivity for detecting *T. solium* cysts than the previous two methods (31, 33, 34). However, carcass dissection is not feasible as a routine method for detecting cysts because of the destruction of a valuable commercial commodity and time constraints (31).

In addition to medical imaging in humans and the more ‘physical’ examination forms described above for pigs, a wide range of serological diagnostic methods have been published. Given the varied performance of the published serological tests, analyzing trends and singling out the best tests is difficult. To address this, we conducted a comparative analysis of the performance of serological tests used for NCC and PCC diagnosis published from 2000 to 2024, based on *T. solium*-specific IgG detection. The published literature in PubMed^1^ was searched with keywords “*Taenia solium*”, “neurocysticercosis”, “pig/porcine cysticercosis”, “serodiagnosis” with the Boolean operator AND, which identified relevant serology diagnostics-based studies. Studies which did not report both sensitivity and specificity were not included. Here, we discuss the various serological tests used for the diagnosis of cysticercosis, in humans and pigs, along with their limitations, and compare their sensitivity and specificity. We also describe the prospects of developing an IgE-based serological test to diagnose *T. solium* infections.

## 2 Available serological tests used for *Taenia solium*

Serological tests are the essential diagnostic tools in low-and middle-income countries (LMIC) for human and pig *T. solium* cysticercosis. These tests are based on detecting circulating *T. solium* antigens or anti-*T. solium* antibodies in serum (35), cerebrospinal fluid (CSF) (36), saliva (37) or urine (38). Serodiagnosis of cysticercosis through the detection of antibodies produced against *T. solium* has been based on different target antigenic preparations, ranging from total *T. solium* cysts extracts to more selected preparations, such as cyst fluid, scolex extract, or tegumental extract (39, 40). Additionally, lentil lectin-purified glycoproteins (LLGP) (41), recombinant and recombinantly expressed antigens (39, 42), monoclonal antibodies (mAb) (43, 44) of *T. solium* have been evaluated for serological diagnosis of cysticercosis. A critical issue is that existing serological tests show high levels of cross-reactivity with other taeniids in the field (45).

The two primary serological methods currently used for diagnosing cysticercosis in humans and pigs are the enzyme-linked immunosorbent assay (ELISA) and the enzyme-linked immune electro-transfer blot (EITB) based on lentil lectin-purified glycoproteins (LLGP) antigens and/or recombinant and synthetic antigens (12, 45, 46).

Antibody-capture ELISA techniques (Ab-ELISA) identify circulating *T. solium* antibodies in serum or CSF, indicating *T. solium* exposure in a population. The antibody-based EITB (LLGP) immunoblot was first introduced in 1989 based on seven diagnostic LLGP proteins of *T. solium* (41). In this assay, EITB combines sodium dodecyl sulfate-polyacrylamide gel electrophoresis (SDS-PAGE) and ELISA techniques to identify specific anti-*T. solium* antibodies in serum or CSF (45). This immunoblot method can detect antibodies to one or more of the seven LLGP antigens (GP 50 family-GP50; T24 family-GP42-39, GP24; 8 kDa family-GP21, GP18, GP14, and GP13) (46). The EITB (LLGP) assay is available through the CDC Parasitic Disease Reference Laboratory for clinical diagnosis of NCC in the United States (12). Recombinant EITB based on recombinant antigens of LLGP also showed promising results for diagnosing PCC/NCC (47, 48). EITB-based LDBIO cysticercosis Western blot kit (LDBIO, Lyon, France) has been developed to identify anti-*T. solium* IgG antibodies in human sera for diagnosing cysticercosis and NCC in humans (49). Furthermore, active/viable infection can be determined by detecting circulating *T. solium* cysticercus antigens using a monoclonal antibody (mAb)-based antigen-capture ELISA (Ag-ELISA). The HP10 (43) and B158/60 (44) mAbs are mostly used to detect *T. solium* infections in humans and pigs. The B158/B60 Ag-ELISA has been commercialized (apDia, Belgium) in the United States (12). Recently, the World Health Organization (WHO) has acknowledged a rapid test for cysticercosis detection that can be performed at the point of care (POC) (50). This rapid POC test or lateral flow assay (LFA) has been developed experimentally based on cyst fluid (51), recombinant antigens (52–56) or mAbs (57, 58) of *T. solium* using human serum/CSF/urine sample. In LFA, the liquid sample is placed on a solid phase-based platform (porous paper, microstructured polymer) that can transport fluid. The sample is run along the surface of the platform with reactive molecules that show a visual positive or negative result. The results are displayed within 5–30 min (59).

## 3 Pig cysticercosis (PCC) serodiagnosis

Serological assays for PCC have shown a higher sensitivity than traditional tongue palpation and carcass/meat inspection (31, 34, 60). The Ab-ELISA test was initially developed using antigens from crude worm extracts, cyst fluids, and excretory-secretory products of taeniid parasites. These unpurified complex antigenic mixtures have shown low sensitivities in Ab-ELISA-based diagnosis (35–67%) of PCC (32, 61, 62). EITB (LLGP) has initially reported 100% sensitivity and specificity in diagnosing PCC (63). However, the same test showed moderate sensitivity (88.9%) and unacceptably low specificity (48.3%) for identifying PCC under field conditions in Peru (64). This type of low specificity may be due to the cross-reaction of the GP50 band of the LLGP protein with *T. hydatigena* or other taeniids (65, 66). A recombinant *T. solium* antigen (rTs-p27)-based EITB was used to test naturally infected pigs in Mozambique, and the results showed disappointingly low sensitivity (29.7%) and specificity (71.7%) (67). EITB can produce more consistent results when the pigs have more cysts, and even in the absence of viable cysts, the test showed good reactivity if the pigs have a relatively large number of non-viable cysts (64). The sensitivity of the EITB (LLGP) assay has been compared with the Ag-ELISA test based on HP10 and B158/60 mAbs in a community-based study of pigs in South Africa. The results identified comparable sensitivities and specificities for B158/B60 Ag-ELISA (76.3 and 84.1%, respectively), HP10 Ag-ELISA (54.8 and 83.3%, respectively), and EITB (LLGP) (45.3 and 85.3%, respectively) (68). The apDia cysticercosis antigen (B158/B60) detection test showed 100% sensitivity (*n* = 31) and 99.6% specificity (*n* = 300) in experimentally infected pigs (69). B158/B60 Ag-ELISA showed similar sensitivity (82.7 and 82.9%) but variable specificity (86.3 and 96.8%) in naturally infected pigs of Tanzania and Peru, respectively (35, 70). In recent years, LFA has improved, using new technology in signal-amplification strategies, applications of new labels, improved quantification systems, and simultaneous detection (59). For instance, up-convert phosphor nanoparticles-based LFA was used for PCC. In this study, TSOL18 (oncospheral stage protein, the antigen used for vaccine development) and GP50 (cystic stage protein) antigens achieved higher sensitivity (93.5 and 97.4%, respectively) and specificity (100% for both) as compared to ELISA. Besides, the cystic larvae were significantly distinguished from those of *T. asiatica*, *Toxoplasma gondii*, *Clonorchis sinensis*, and *Trichinella spiralis*, suggesting a possible higher specificity of the test strip (71). However, this study did not include *T. hydatigena* and *Echinococcus*, which are important candidates for cross-reactivity in PCC diagnosis.

## 4 Human cysticercosis (HCC) serodiagnosis

*Taenia solium* larvae are capable of infecting both neural and extraneural tissues in humans. While human cysticercosis (HCC) predominantly involves the central nervous system (neurocysticercosis), dissemination to extraneural tissues can also occur, occasionally presenting as isolated cysts in sites such as muscle, subcutaneous tissue, and other organs (25, 26, 72, 73). Immunologically, extraneural cysticercosis is characterized by elevated levels of various immunoglobulins, including IgM, IgA, IgE, and IgG (74). Ab-ELISA based on IgG has been reported to diagnose pulmonary and intramuscular cysticercosis through serum samples, offering a supportive diagnostic tool in conjunction with imaging (25, 75). In a population-based study in Brazil, Moraes et al. (76) utilized Ab-ELISA to detect antibodies against *T. solium* metacestodes in both children and adults. The study reported a relatively high seroprevalence, attributed to cross-reactivity and prior exposure rather than active infection, highlighting a common limitation of IgG-based serological assays in endemic areas (76). Notably, IgA-ELISA has demonstrated 100% sensitivity in detecting IgA antibodies in tear samples for the diagnosis of ophthalmic cysticercosis in humans (77).

## 5 Human neurocysticercosis (NCC) serodiagnosis

NCC is known to result in different antibody response patterns during various phases of the disease (viable/active and inactive/degenerated cyst stage) (78). These variations in immune response have a significant impact on the diagnostic ability of serological tests. Ab-ELISA has been used for NCC diagnosis using *T. solium* antigens and recombinant antigens. Bueno et al. (79) reported that Ab-ELISA with *T. solium* total antigen for any NCC achieved 80% sensitivity and specificity in serum samples, whereas CSF samples yielded higher diagnostic accuracy, with 100% sensitivity and 90% specificity. Cyst fluid antigen-based Ab-ELISA were used in some studies, which demonstrated variability in diagnostic performance, with sensitivity ranging from 80 to 100% and specificity from 75 to 96% (80–83). Sahu et al. (84) used both somatic and excretory-secretory (ES) antigens from *T. solium* metacestode. Their findings indicated superior performance with CSF-derived ES antigens, yielding 88.2% sensitivity and 96.9% specificity (84). The recombinant antigens have further improved serodiagnostic accuracy. Lee et al. (85) evaluated an Ab-ELISA based on the recombinant *T. solium* metacestode protein rTsM10, which showed high sensitivity (94.3%) and specificity (96.4%) in both serum and CSF, particularly useful for detecting early-stage infections. Moreover, recombinant and synthetic antigens such as rT24H, rGP50, and Ts18var1 have been incorporated into the QuickELISA^™^ diagnostic platform to compare the performance of selected antigens. Among these, T24H QuickELISA^™^ showed the highest diagnostic performance, with a reported sensitivity of 96.3% and specificity of 99.2% (86). These findings were supported by Hernández-González et al. (87), who reported similar diagnostic accuracy using rT24H. Lee et al. studied low-molecular-weight proteins (ranging from 7–38 kDa) in *T. solium* cyst fluid for Ab-ELISA (88). These proteins were analysed from different geographical regions (Korea and Mexico) and showed diagnostic sensitivity and specificity of 97.7 and 98.7%, respectively, against the extraparenchymal NCC (89). The immunoblot, EITB (LLGP), showed 100% specificity and 95% sensitivity in patients with multiple active cysts, including intracranial lesions (41, 78, 90, 91). The GP50 band of EITB (LLGP) appeared first with initial exposure or cyst stage of NCC and remained even after the cyst resolution stage, while T24 responses are heterogeneous (46). Any response against the 8 kDa family indicated active infections or high antigen burden (78, 92). Recombinant and synthetic antigens (rT24H, rGP50, and sTsRS1)-based EITB showed very high sensitivity (99%) and specificity (98%) in patients with ≥2 viable cysts (48). This recombinant EITB method remained negative when they tested with hydatid echinococcosis positive sera, although the technique showed lower sensitivity for detecting NCC cases with a single viable cyst (56%, CI: 40–67) and a calcified cyst (78%, CI: 68–87). Handali et al. (93) developed a multiantigen printing immunoassay (MAPIA) based on six EITB diagnostic protein families (rGP50, rT24H, and peptides sTsRS1, sTs18var1, sTsRS2var1, and sTs14) which reached 97 and 99% sensitivity and specificity with both intra-and extraparenchymal NCC. Another MAPIA-based study using 3 diagnostic proteins (rGP50, rT24H, and sTs14) detected 97.5% parenchymal NCC cases and 100% subarachnoid cases, with 98.53% specificity (94). Tang et al. (95) reported a multiple triplex ELISA using rT24H, rGP50 and sTs18var3 in EITB-format; the highest sensitivity and specificity were observed for rGP50 (94 and 98%) with lower sensitivities obtained with rT24H (81, 92%) or sTs18var3 (93, 92%). A multiplex bead assay (MBA) based on two recombinant proteins (rT24H, and rTs8B2) also showed high sensitivity and specificity (96.1 and 96.5%, respectively) in cases with two or more viable cysts (87). However, in one study, five commercially available *T. solium* ELISA diagnostic tests (DRG^™^, Ridascreen^™^, Novatech^™^, Cypress^™^, and IVD^™^), developed from native or less purified versions of total and vesicular antigen of metacestodes, showed variable low sensitivity (42.9, 71.4, 42.9, 50, 42.9%) and specificity (93.7, 74.2, 95.6, 86.8, 90.6%), respectively. The result showed cross-reaction with all *Echinococcus granulosus*-positive sera and false-positive reactions to *E. multilocularis* positive sera. All the tests detected cross-reactions with other unrelated parasites such as *Entamoeba histolytica*, *Schistosoma* sp., *Fasciola hepatica*, *Strongyloides stercoralis*, *Trichinella* sp., and filarial worms (89).

Ag-ELISA has been reported as a highly sensitive and specific test for *T. solium* infections in humans with viable cysts (≥2) (96). The sensitivity of NCC diagnosis in humans using mAb-based HP10 and B158/B60 Ag-ELISA depends on the location of the cyst lesions. They can detect extra-parenchymal cysts more easily than intra-parenchymal cysts (15, 97). Moreover, these assays were reported to help support the diagnosis of NCC using different samples like CSF (36, 98), serum (97, 99), and even urine representing a non-invasive sampling approach (38, 46, 100). HP10 Ag-ELISA showed higher sensitivity (94.1%) and specificity (97.7%) in NCC patients with multiple viable cysts and inflammatory disease. However, the study was less sensitive (33.3%) for patients with non-inflammatory conditions and single viable cysts (36). The manufacturer of the commercialised B158/B60 Ag-ELISA (apDia, Belgium) reports a sensitivity of 94% (*n* = 100) and specificity of 99.3% (*n* = 300) (69). In one study, B158/B60 Ag-ELISA showed sensitivity and specificity of 100.0 and 84.0%, respectively, in diagnosing active infections in people with epilepsy (99). Two mAbs (TsW8 and TsW5) have currently been explored for accurately detecting antigen levels in serum and urine of NCC patients (46, 101). In other studies, QuickELISA^™^ was developed using rT24H, rGP50, and sTs18var1. The results identified better sensitivity and specificity with two or more viable cysts for rT24H (92.6, 97.8%) than rGP50 (89.8, 97.5%) and sTs18var1 (84.3, 93.4%) (86). This QuickELISA^™^ is a faster alternative to conventional ELISA. It relies on using two antigen conjugates, antigen-streptavidin and antigen-horseradish peroxidase, to capture and detect specific antibodies instead of secondary enzyme-labelled conjugates (102). One study reported 97% sensitivity and 95% specificity using single-chain viable fragments of antibodies (G10, A4, and B6), produced by phage display against *T. solium* total saline extraction (103). IgM-secreting hybridomas specific to *T. solium* (100) have been converted to mouse IgG, and resulted in a recombinant mAb (TsG10) with the highest affinity to crude *Taenia* antigen. The test achieved a 98% sensitivity and 100% specificity in detecting extraparenchymal NCC from serum, plasma, and CSF of patients. This assay could decrease the cost of mAb production and might be a more affordable option to detect circulating antigens (104). The good performance of the mAb-based Ag-ELISA test has initiated the development of LFA. Recombinant *T. solium* antigen (rT24H)-based LFA showed a good response (93.9% sensitivity and 98.9% specificity) for NCC diagnosis with two or more viable brain cysts (102). HP10 Ag-LFA showed high sensitivity and specificity (100%) using a CSF sample for detecting extraparenchymal NCC (57), but low or negative levels were observed in the CSF and serum samples from patients with parenchymal NCC (14). Quantum dots (Qdots)-labelled LFA reported 89% sensitivity and 99% specificity for identifying specific antibodies in sera of infected humans (105). The *T. solium* diagnostic project (SOLID) developed a two-strip *T. solium* point of care test (TS POC) prototype for the detection of antibodies against the adult stage (rES33), and larval stage (rT24H) of the parasite (56). This POC test was evaluated in two endemic countries, Zambia and Tanzania. In Zambia, the results showed low sensitivity and specificity (35 and 87%) in NCC patients as compared to rT24H-EITB (94 and 95%) and B158/B60 Ag-ELISA (36 and 87%) (53). In Tanzania, Stelzle et al. (54) compared the TS POC test with rT24H-EITB, LLGP-EITB, and Ag-ELISA and reported a sensitivity of 49% and a specificity of 91% for any type of NCC. The sensitivity of the TS POC test was comparable to Ag-ELISA (50%) and rT24H-EITB (44%) and higher than LLGP-EITB (23%). The researchers reported this test as very sensitive for NCC patients with vesicular lesions (54). LDBIO developed a commercial EITB kit called CYSTICERCOSIS Western Blot IgG (LDBIO, Lyon, France) for detecting HCC and NCC (49). Salazar-Anton et al. (106) compared the effectiveness of four immunodiagnostic techniques to diagnose NCC. The serum was tested with a commercial LDBIO EITB kit (Lyon, France) and later tested with an in-house immune-dot blot (107) with Tsol-p27 antigen, Ag-ELISA, and Western blot with Tsol-p27/TsolHSP36 antigens. The result showed the highest sensitivity and specificity for immunodot blot-Tsol-p27 (86.7 and 97.8%) compared to Ag-ELISA (86.7, 94.6%), and Western blot with Tsol_p27 (76.4, 95.6%) and Tsol HSP36 (61.9, 86.1%). A recent POC test based on TsW8/TsW5 mAbs in urine detected 97% of extraparenchymal NCC patients with a specificity rate of 100%. This rapid test can be performed in 15 min and could be a good option for low-resource areas (58). However, additional scrutiny is required for cross-reactivity with related parasites such as *Echinococcus* sp., which are frequently co-endemic with cysticercosis.

The differences in outcomes of serodiagnosis of NCC can be influenced by several factors, which may include the stage of *T. solium* infection, choice of antigens for serological assays, design of the study, location of cyst lesions in the brain, number of lesions, transient immunity among patients in endemic countries (34, 54), cross-reactivity with other parasite antigens, and prevalence and genetic variation across different endemic countries. The inclusion or omission of the close relative genus *Echinococcus* spp. in the study (as mentioned above), use of serum/CSF sample, and using sera with previously positive serology may be other potential influencing factors for human cysticercosis diagnostic test results, complicating their comparison and interpretation.

## 6 Limitations and critical appraisal

The available serological tests have shown significant variability in their sensitivity and specificity for diagnosing cysticercosis in humans and pigs (Table 1). ELISA is critical for identifying cysticercosis in endemic countries as it is cheaper (US$5-US$30/test) and more affordable than neuro-imaging (34, 65). However, the limitation of these tests is cross-reactivity with other taeniid parasites, particularly in cases where the diseases are co-endemic; furthermore, antibody detection tests cannot distinguish between active and inactive, past infections (108). The commercial apDia ELISA test for NCC has shown decreased sensitivity when the number of viable cysts is low, and cannot detect infections involving a single viable cyst. For PCC, the test does not allow the differentiation between infections of different *Taenia* species in pigs and is not cost-effective (€3.15/sample) (34). This specificity problem is significant in regions where *T. hydatigena* (Sub-Saharan Africa, South East Asia) (109, 110) and *T. asiatica* (Southeast Asia) (111) are prevalent alongside *T. solium* (69). Although EITB is widely preferred to detect anticysticercal antibodies, it is not field-friendly, not widely available, and does not exist commercially in point-of-care formats (102). In addition, EITB does not confirm central nervous system infection since it demonstrates a systemic antibody response (24) and has insufficient sensitivity with single intracranial cysts and calcified parasites (112, 113). In experimental studies, LFA tests have shown promising results for cysticercosis diagnosis; however, they are currently unavailable at commercial levels and can only detect active, viable cysts (4, 45, 93).

**Table 1:** Serological tests used for cysticercosis diagnosis in humans and pigs with estimated sensitivity and specificity.

Diagnostic tools	References	Hosts	Sample	Test materials	Uniprot ID & molecular weight/related information	Sensitivity (sens)	Specificity (spec)	Comments
Ab-ELISA	Bueno et al. (79)	Human	Serum	Ts total Ag		80 (72–88)	80 (73.2–86.8)	60% samples with active NCC
			CSF	Ts total Ag		100 (99.4–100)	100 (99–100)	
	Bueno et al. (80)		Serum	Ts cyst Ag		100	90	Sample with confirmed or suspected NCC
	Gekeler et al. (81)		Serum/CSF	TsM cyst fluid Ag		80	75.3	Cross-reaction, number of cyst/cyst lesions not included
	Lee et al. (85)		Serum/CSF	rTsM10/m13hv1	Q9U8G9, 9.65 kDa	94.3	96.4	Samples from confirmed NCC patients; single cyst not included
	Arruda et al. (82)		Serum	TsM cyst fluid Ag		91.3	96.2	Samples from confirmed NCC patients
	Oliveira et al. (83)		Serum	TsM cyst fluid Ag		95 (92.8–97.2)	83 (76–90.6)	Samples from confirmed NCC patients
	Sahu et al. (84)		Serum	TsM somatic Ag		76.4	97.9	Degenerated stage of NCC
				TsM ES Ag		88.2	96.9	Live vesicular stage of NCC
			CSF	TsM somatic Ag		75	96.4	Degenerated stage of NCC
			CSF	TsM ES Ag		64.2	97.6	Live vesicular stage of NCC
	Lee et al. (88)		Serum	TsM-LMWPs		90.6	98.7	90% samples with active NCC
				TsM cyst fluid Ag		97.7	83.7	Cross reactions with other parasites
	Lee et al. (86)		Serum	rT24H	Q5GM22, 23.91 Kda	96.3	99.2	Sens data of two or more viable cysts
				rGP50	Q6XR69, 32.78 kDa	93.5	98.6	Sens data of two or more viable cysts
				Ts18var1	Q8T7L3, 8.83 kDa	89.8	96.4	Sens data of two or more viable cysts
	Hernández-González et al. (87)		Serum	rT24H	Q5GM22, 23.91 kDa	88.5	99.4	Sens of single viable cyst
	Nunes et al. (61)	Pigs	Serum	Ts cyst fluid Ag		67.8	98.2	Field-level study
	Pouedet et al. (62)		Serum	TsM cyst fluid Ag		68.3	84.4	Field-level study
	Dorny et al. (32)		Serum	Tcra cyst fluid Ag		35.8 (26–41)	91.7 (85–99)	Pigs with light infection, crude Ag
	Assana et al. (127)		Serum	F3, cyst fluid Ag		58.5 (42.5–78.7)	75.4 (68.9–81.8)	Field-level study
	Ramahefarisoa et al. (128)		Serum	TsM cyst fluid Ag		100	73	Heavily infected pigs (>5 cysts), cross-reaction
	Sreedevi et al. (129)		Serum	TsM cyst fluid Ag		96	96	Sample with confirmed pig cysticercosis
				TsM whole cyst Ag		96	92	Sample with confirmed pig cysticercosis
EITB	Aguilar-Rebolledo et al. (130)	Human	Serum/CSF	LLGP-7 Ag	50, 42-39, 24, 21, 18, 14, 13 kDa	59	96	Sens and spec of single viable cyst
	Gekeler et al. (81)		Serum/CSF	TsRS1, TsTrx-1, Ts26GST	Q8ST31, 8.6 kDa; A0A0A1E6L3, 11.55 kDa; Q8MWS0, 25.49 kDa	81.7	99.4	Samples from confirmed NCC with non-endemic controls
	Noh et al. (48)		Serum	rT24H	Q5GM22, 23.91 kDa	52 (41–62)	98 (96–100)	Sens and spec of single cyst
				rGP50	Q6XR69, 32.78 kDa	44 (31–58)	100 (100)	
				TsRS1	Q8ST31, 8.6 kDa	39 (28–49)	100 (100)	
	Dermauw et al. (131)		Serum	rT24H	Q5GM22, 23.91 kDa	66.7 (9.42–99.2)	98.2 (93.8–99.8)	Sens and spec of single viable cyst
				rGP50	Q6XR69, 32.78 kDa	100 (29.2–100)	98.2 (93.8–99.8)	
				rT24H + rGP50	Q5GM22, 23.91 kDa; Q6XR69, 32.78 kDa	100 (29.2–100)	98.2 (93.8–99.8)	
	Mubanga et al. (53)		Serum	rT24H	Q5GM22, 23.91 kDa	94 (91–98)	95 (91–98)	Samples from confirmed cysticercosis by TS POC cysticercosis test
	Jiménez et al. (132)		Serum	Ts26GST	Q8MWS0, 25.49 kDa	79 (65–91)	86 (76–96)	Recognized non-viable and viable cyst, 70% single cyst case detected
				TsTrx-1	A0A0A1E6L3, 11.55 kDa	88.6 (79–98)	98 (94–100)	
	Tang et al. (95)		Serum	GP50	Q6XR69, 32.78 kDa	94 (88–97)	98 (90–100)	Most of the patients with extraparenchymal NCC
				Ts18var3	Q9U562, 8.41 kDa	93 (86–97)	92 (82–97)	
				T24H	Q5GM22, 23.91 kDa	81 (72–88)	92 (82–97)	
	Stelzle et al. (54)		Serum	LLGP-7 Ag	50, 42–39, 24, 21, 18, 14, 13 kDa	23 (18–28)	95	Sens and spec of any type of NCC
			Serum	rT24H	Q5GM22, 23.91 kDa	44 (37–51)	98	
	Zulu et al. (55)		Serum	rT24H	Q5GM22, 23.91 kDa	23 (8–48)	89 (79–94)	Sens and spec of any type of NCC, field level study
	Krecek et al. (133)	Pigs	Serum	LLGP-7 Ag	50, 42–39, 24, 21, 18, 14, 13 kDa	49 (36.4–62.8)	84 (75–91.8)	Sample with more active infection in pig
	Jayashi et al. (64)		Serum	LLGP-7 Ag	50, 42–39, 24, 21, 18, 14, 13 kDa	88.9 (65.3–98.6)	48.3 (37.6–59.1)	Sens and spec of single reactive bands of EITB
	Nhancupe et al. (67)		Serum	p27	F6M8L6, 26.71 kDa	29.7	71.7	Sample from naturally infected pigs, cross-reaction
	Gupta et al. (134)		Serum	Crude TsM Ag		93.3 (77.9–99.1)	100 (92.9–100)	Samples confirmed for PCC by MRI
Ag-ELISA	Garcia et al. (96)	Human	Serum	HP 10	*T. saginata* metacestode antigen	85	92	Samples seropositive for NCC on EITB
	Fleury et al. (36)		CSF	HP10	*T. saginata* metacestode antigen	100	97.7	Sens of multiple cyst NCC
	Nguekam et al. (135)		Serum	B158/B60	B158C11A10 and B60H8A4 IgG1 mAb against ES products of *T. saginata*	94.4	100	Sample from endemic areas; active cysticercosis
	Fleury et al. (15)		Serum	HP 10	*T. saginata* metacestode antigen	84.8 (74.4–95.2)	94 (90.2–97.8)	Samples from radiologically and clinically NCC patients
	Castillo et al. (38)		Urine	B158/B60	B158C11A10 and B60H8A4 IgG1 mAb against ES products of *T. saginata*	62.5	100	Sens of single cyst
	Gabriël et al. (99)		Serum	B158/B60	B158C11A10 and B60H8A4 IgG1 mAb against ES products of *T. saginata*	100 (61–100)	84 (75–91)	Samples from people with epilepsy (PWE)
	Fleury et al. (57)		CSF	HP 10	*T. saginata* metacestode antigen	100	100	All Samples from extraparenchymal NCC
	Mubanga et al. (53)		Serum	B158/B60	B158C11A10 and B60H8A4 IgG1 mAb against ES products of *T. saginata*	36 (15–67)	87 (81–92)	Sample with low prevalence of human cysticercosis
	Stelzle et al. (54)		Serum	B158/B60	B158C11A10 and B60H8A4 IgG1 mAb against ES products of *T. saginata*	50 (43–56)	97	Sens and spec of any type of NCC
	Zulu et al. (55)		Serum	B158/B60	B158C11A10 and B60H8A4 IgG1 mAb against ES products of *T. saginata*	30 (11–58)	82 (71–89)	Sens and spec of any type of NCC, field-level study
	apDia (Belgium), Ref. 650,501 (69)		Serum/CSF	B158/B60	B158C11A10 and B60H8A4 IgG1 mAb against ES products of *T. saginata*	94 (87.4–97.8)	99.3 (97.6–99.9)	Commercial test kit for cysticercosis, lab-based sens and spec
	Pouedet et al. (62)	Pigs	Serum	B158/B60	B158C11A10 and B60H8A4 IgG1 mAb against ES products of *T. saginata*	85.8	98.9	Field-level study
	Dorny et al. (32)		Serum	B158/B60	B158C11A10 and B60H8A4 IgG1 mAb against ES products of *T. saginata*	86.7 (62–98)	94.7 (90–99.7)	Sample with degenerated cyst, field-level study
	Assana et al. (127)		Serum	B158/B60	B158C11A10 and B60H8A4 IgG1 mAb against ES products of *T. saginata*	89.5 (80.4–99.4)	94.7 (90.2–99.7)	Field-level study
	Krecek et al. (133)		Serum	HP 10	*T. saginata* metacestode antigen	70.4 (52.7–84.7)	66.1 (44.6–85.1)	Cross-reactions
				B158/B60	B158C11A10 and B60H8A4 IgG1 mAb against ES products of *T. saginata*	63.3 (46.8–81.6)	87 (78.2–94.9)	Inactive infections in pig
	Chembensofu et al. (136)		Serum	B158/B60	B158C11A10 and B60H8A4 IgG1 mAb against ES products of *T. saginata*	91 (71–99)	67 (47–83)	Sens of viable cysts (1 or more)
	Bustos et al. (35)		Serum	B158/B60	B158C11A10 and B60H8A4 IgG1 mAb against ES products of *T. saginata*	82.9 (69.7–96)	96.8 (90.2–100)	Not considering cross-reactions with *T. hydatigena*
	Kabululu et al. (70)		Serum	B158/B60	B158C11A10 and B60H8A4 IgG1 mAb against ES products of *T. saginata*	82.7 (64.2–94.1)	86.3 (82–89.9)	Samples tested by necropsy, optimal sens in > 50 cysts
	apDia (Belgium), Ref. 650,501 (69)		Serum	B158/B60	B158C11A10 and B60H8A4 IgG1 mAb against ES products of *T. saginata*	100 (83.8–100)	99.7 (98.2–99.9)	Commercial test kit for cysticercosis, lab-based sens and spec
LFA	Handali et al. (102)	Human	Serum	rT24H	Q5GM22, 23.91 kDa	93.9	98.9	Sens and spec of two or more viable brain cysts
	Fleury et al. (57)		CSF	HP 10		100	100	All Samples from extraparenchymal NCC
	Lee et al. (105)		Serum	rT24H	Q5GM22, 23.91 kDa	44 (22–69)	99 (95–100)	Sens of single viable cyst
	Mubanga et al. (53)		Serum	rT24H + rES33	Q5GM22, 23.91 kDa; Q6EMI6, 29.19 kDa	35 (14–63)	87 (83–90)	Field-level study
	van Damme et al. (52)		Blood	rT24H	Q5GM22, 23.91 kDa	24.9–77.5	92.3–99.1	Different populations used for sampling, field level study
	Stelzle et al. (54)		Serum	rT24H + rES33	Q5GM22, 23.91 kDa; Q6EMI6, 29.19 kDa	49 (41–58)	91 (89–94)	Sens and spec of any type of NCC
	Zulu et al. (55)		Serum	rT24H	Q5GM22, 23.91 kDa	26 (15–41)	88 (85–90)	Sens and spec of any type of NCC, field level study
	Toribio et al. (58)		Urine	B158/B60	B158C11A10 and B60H8A4 IgG1 mAb against ES products of T. saginata	96.7	100	Samples from patients with subarachnoid NCC (high Ag level)
	Zhang et al. (71)	Pigs	Serum	Tsol18	A8Y989, 18 kDa	93.4	100	Using positive serum of pig, not including *T. hydatigena*
				GP50	Q6XR69, 32.78 kDa	97.4	100	

**Figure 2:**
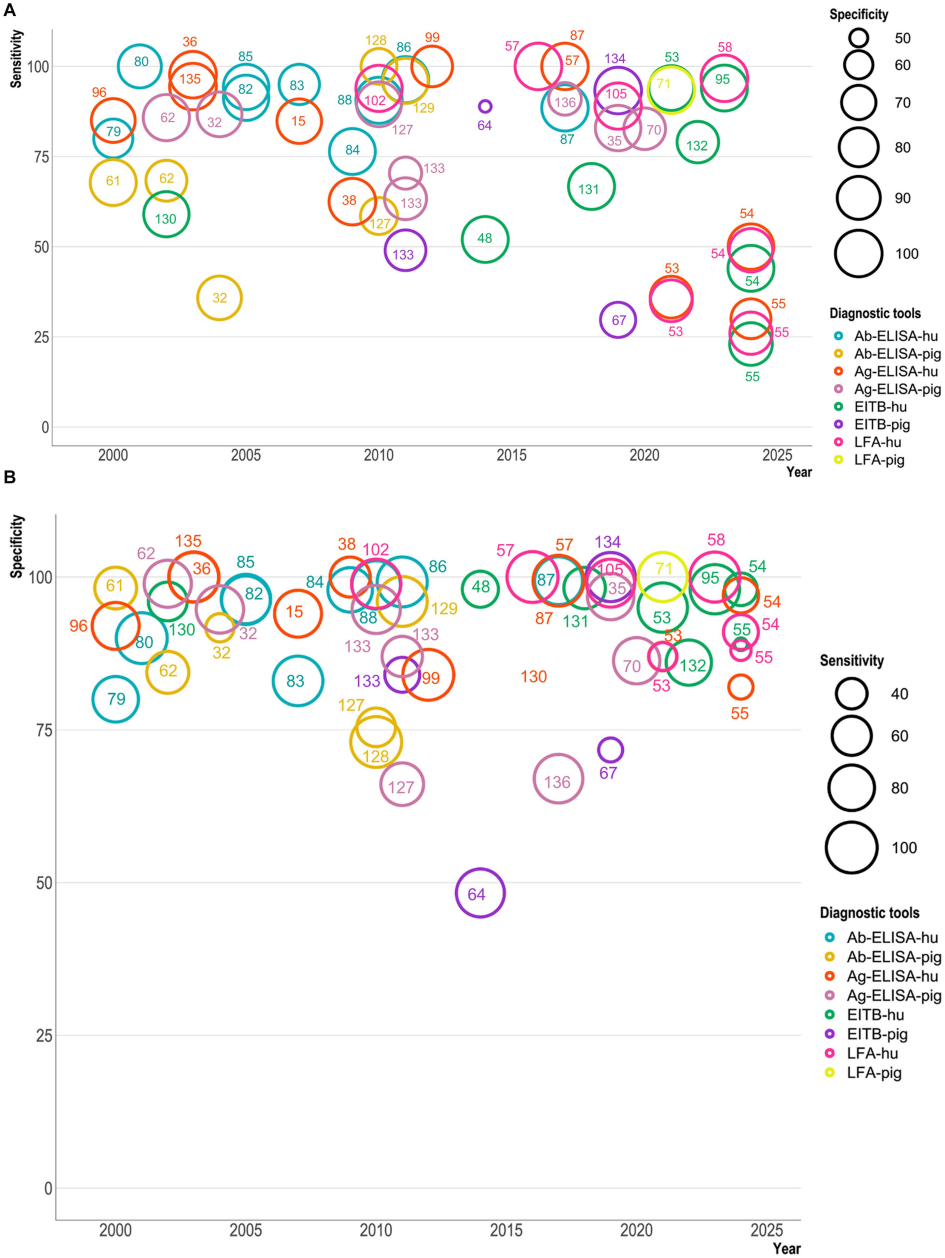
A bubble plot showing sensitivity and specificity of serological diagnostic tests for *Taenia solium* (Y-axis) published from 2000–2024 (X-axis). **A** represents the sensitivity plot, while **B** shows the specificity plot. The bubble size in **A** indicates the test’s specificity, while in **B**, it indicates the sensitivity. The color of the bubbles indicates different serological tests used for diagnosing cysticercosis in humans and pigs. The serological tests are Ab-ELISA (antibody ELISA), Ag-ELISA (antigen ELISA), EITB (enzyme-linked immunoelectrotransfer blot), and LFA (lateral flow assay), which have been marked with reference numbers with a test-specific color. These bubble plots include nine Ab-ELISA (79, 80, 82–88), eleven Ag-ELISA (15, 36, 38, 53–55, 57, 87, 96, 99, 135), eight EITB (48, 53–55, 95, 130–132), and seven LFA (53–55, 57, 58, 102, 105) tests for human NCC. In contrast, six Ab-ELISA (32, 61, 62, 127–129), eight Ag-ELISA (32, 35, 62, 68, 70, 127, 133, 136), four EITB (64, 67, 68, 133, 134), and one LFA (71) test have been used for PCC.

In an effort to visualize trends underlying the development of the different serological techniques used, we used R software^2^ to create a bubble plot that compares the year of publication of the serological tools and the sensitivity/specificity of the test (Figure 2). This representation allows us to represent four parameters: sensitivity, specificity, year of publication, and the serodiagnosis technique used for humans or pigs.

Our bubble plot analysis show that in serodiagnosis, sensitivity has not steadily improved over the years and has produced more scattered percentages (even ≤30%) than specificity percentages (≥ 50%) of tests. In Figure 2A, the sensitivity of serodiagnosis tests ranged between 25 and 100%, with few tests showing lower results (less than 25%). Ag-ELISA and Ab-ELISA tests have shown variability in their sensitivity and specificity. Ag-ELISA for humans showed less sensitivity (25–100%) than for pigs (50–100%). EITB tests for humans and pigs showed the lowest sensitivity (≤25–30%). The POC/LFA test sensitivity varied between 25 and 100% for humans, while one LFA study showed >90% sensitivity in pigs. In the specificity plot (Figure 2B), most serodiagnosis tests’ specificity ranges between 70 and 100%. Ab-ELISA and Ag-ELISA for humans and pigs showed 80–100% specificity, while one Ag-ELISA test showed around 70% specificity for pigs. EITB for humans has shown higher specificity (89–100%), but for pigs, the specificity is as low as around 50%. LFA for humans showed the best specificity, although sensitivity (even around 25%) is a problem. It is essential to note that not all studies included echinococcosis serum samples for human or other co-endemic *Taenia* species of pigs, which may have resulted in some cases with higher specificities than in field studies, as species-specific resolution of *Taenia* infection and distinction from Echinococcosis are notoriously challenging to achieve serologically.

## 7 Serodiagnosis of cysticercosis: investigating an IgE-based approach?

*T. solium* infection in humans and pigs induces specific antibody responses, especially Immunoglobulin G (IgG), and as discussed above, has been frequently used for serological diagnosis of cysticercosis. However, most metazoan parasitic infections typically also result in elevated levels of total and parasite-specific serum IgE in the infected host (114). IgE antibodies induce a protective immune response against invading parasites by initiating a Th2-biased response involving the high-affinity IgE receptor (FcεRI) (mast cells, basophils, dendritic cells, eosinophils) and the low-affinity IgE receptor (CD23) (eosinophils, dendritic cells, platelets, macrophages, and B cells) (115, 116).

NCC in humans is often associated with higher levels of parasite-specific antibodies (IgG1, IgG2, IgG4, IgE) (117). Flisser et al. (118) identified all immunoglobulin classes (IgG, IgM, IgE, IgA, and IgD) in decreasing order in NCC patients in serum, while Espinoza et al. (119) found IgG, IgA, and IgE in CSF. Cerebral cysticercosis has been reported with increased IgE levels in CSF or serum in infected patients (120). In Brazil, one study found elevated total IgE levels among all individuals with anti-*T. solium* cysticercus antibodies (42). Another group used *T. solium* and *T. crassiceps*-based Ag-ELISA to measure and compare immunoglobulins present in serum and CSF samples of NCC patients, in which IgE detection showed 24% sensitivity and 97.1% specificity for serum but was negative in CSF samples. This study also reported that IgE was more frequent in the patients with the inactive cyst form, with no degenerating cysts or immune-inflammatory processes (79). However, using the same method, Espinoza et al. (119) detected only 3% positivity for IgE in serum and CSF of NCC patients. For pigs, *T. solium* cysts showed detectable serum IgG levels in experimentally infected animals after 30 days of egg inoculation (60). However, IgE levels in PCC have not been studied yet.

Transgenic humanized reporter systems are extremely sensitive and can detect as little as 15 pg./mL of IgE because of their intrinsic signal amplification mechanisms (121). Using this reporter system may improve the early detection of cysticercosis (122). In human experimental infection with the hookworm *Necator americanus*, using basophil activation tests, we were able to detect binding of parasite-specific IgE to peripheral blood basophils as soon as 5–6 weeks after human infection, even in the absence of detectable amounts of free parasite-specific IgE in serum (123). This suggests that metazoan parasites with tissue migratory larval stages may be able to induce an early IgE response even in primary infections, possibly or in part due to the release of proteases during their migration (124). Combined with the high sensitivity of IgE reporter systems, this may enable the detection of small amounts of parasite-specific IgE at an early stage of infection. The diagnostic potential of parasite-specific IgE has also been explored, e.g., for toxoplasmosis (125) and strongyloidiasis (126), showing high sensitivity and specificity for diagnosing these parasitic infections, albeit not in a reporter cell format. One key advantage of using non-homologous reporter cell assays compared to, e.g., ELISA is that competing antibody isotypes (e.g., IgG4) are easily removed during a washing step, leaving only human IgE bound to the surface of the rat reporter cells, which do not bind human IgG. In contrast, for ELISA-type assays, to avoid competition, which will affect sensitivity, it is necessary to remove IgG from the serum samples by affinity chromatography, increasing cost and labour.

## 8 Conclusion

Cysticercosis is still a significant challenge for public health. Diagnosing cysticercosis in humans is progressing steadily, but PCC diagnostic test development is still lagging. The serological diagnosis of cysticercosis needs to be improved to avoid cross-reactivities and false negative results. *T. hydatigena* has been identified as one of the main cross-reactive species for pig cysticercosis serology. There is a lack of cross-reactivity studies on *T. asiatica* in co-endemic countries. The sensitivity of the serological tests still needs to be improved, as it is still unacceptably low in the case of low cyst numbers or calcified, inactive parasite stages. IgE-based serological tests may be a good alternative for laboratory-based diagnosis of cysticercosis. Difficulties persist regarding differential diagnosis of related parasitoses, such as cysticercosis and echinococcosis, particularly in areas where both parasites are co-endemic. Thus, a combination of serological and molecular technologies, such as loop-mediated isothermal amplification (LAMP) or recombinase polymerase amplification (RPA), may remain necessary.

## Footnotes

^1^ http://www.ncbi.nlm.nih.gov/pubmed/

^2^ https://cran.r-project.org/bin/windows/base/R-4.4.2-win.exe
